# RYBP-PRC1 Complexes Mediate H2A Ubiquitylation at Polycomb Target Sites Independently of PRC2 and H3K27me3

**DOI:** 10.1016/j.cell.2012.06.011

**Published:** 2012-06-22

**Authors:** Lígia Tavares, Emilia Dimitrova, David Oxley, Judith Webster, Raymond Poot, Jeroen Demmers, Karel Bezstarosti, Stephen Taylor, Hiroki Ura, Hiroshi Koide, Anton Wutz, Miguel Vidal, Sarah Elderkin, Neil Brockdorff

(Cell *148*, 664–678; February 17, 2012)

In preparing the figures for the above article, we processed digital data scans to remove irrelevant lanes from single-gel images in some instances (Figures 5A, 7B, S5A, S4A, S4B, and S5B) or, in two cases (Figures 5B and S4), presented large numbers of samples from a single experiment run on two gels as a single panel. The presence of splice marks in these figures has been queried by some readers. We now appreciate that we should have indicated the positions of the splicings with a space or line, as per the stated policy of Cell Press journals, particularly in light of the importance of clarity around data integrity.

We present below a representative example of how we should have depicted the relevant panels by reproducing both the original published gel scan for Figure 5A (RING1B pull-down blotted with anti-RING1B antibody) and a revised panel. We also present Figures 5B and S4 as they should have been displayed. The original figures display the correct data and labeling in all cases, and this oversight has no bearing on the interpretation of individual experiments or the manuscript as a whole. We apologize for any inconvenience that our original displays of these data may have caused.

## Figures and Tables

**Figure 5:**
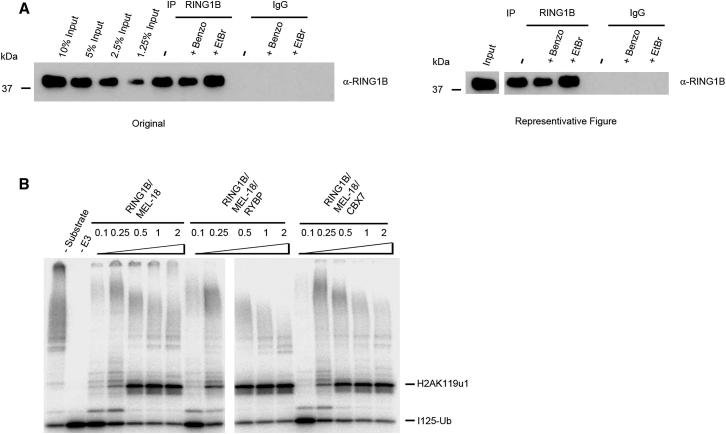
MEL-18 Interacts with RYBP and CBX7 in Mutually Exclusive Catalytically Active Complexes

**Figure S4:**
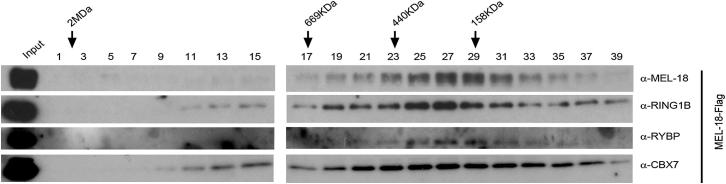
Related to Figure 4

